# The Relationship and Expression of *miR-451a*, *miR-25-3p* and *PTEN* in Early Peritoneal Endometriotic Lesions and Their Modulation In Vitro

**DOI:** 10.3390/ijms23115862

**Published:** 2022-05-24

**Authors:** Warren B. Nothnick, Riley Peterson, Paige Minchella, Tommaso Falcone, Amanda Graham, Austin Findley

**Affiliations:** 1 Department of Molecular and Integrative Physiology, University of Kansas Medical Center, Kansas City, KS 66160, USA; rpeterson6@kumc.edu (R.P.); pminchella@kumc.edu (P.M.); agraham@kumc.edu (A.G.); 2 Department of Obstetrics and Gynecology, University of Kansas Medical Center, Kansas City, KS 66160, USA; afindley@kumc.edu; 3 Institute for Reproduction and Perinatal Research, Center for Reproductive Sciences, University of Kansas Medical Center, Kansas City, KS 66160, USA; 4 Cleveland Clinic, London E1 4DG, UK; FALCONT@ccf.org; 5 Cleveland Clinic, Lerner College of Medicine, Cleveland, OH 44101, USA

**Keywords:** endometriosis, miRNA, miRNA:miRNA interaction, miR-451a, miR-25-3p, PTEN

## Abstract

Background: *miR-451a* can function as a tumor suppresser and has been shown to be elevated in both endometriotic lesion tissue and serum from women with endometriosis. To further explore the role of miR-451a in the pathophysiology of endometriosis, specifically, further evaluating its association with the tumor suppressor, phosphatase and tensin homolog (PTEN), we examined their expression in individual endometriotic lesion tissue to gain insight into their relationship and further explore if *miR-451a* regulates PTEN expression. Methods: A total of 55 red, peritoneal endometriotic lesions and matched eutopic endometrial specimens were obtained from 46 patients with endometriosis. *miR-451a*, *miR-25-3p* and *PTEN* mRNA levels were assessed by qRT-PCR and reported for each matched eutopic and ectopic sample. To evaluate *miR-451a* and *miR-25-3p* expression of *miR-25-3p* and PTEN, respectively, 12Z cells (endometriotic epithelial cell line) were transfected and *miR-25-3p* expression was assessed by qRT-PCR, while PTEN protein expression was assessed by Western blotting. Results: *PTEN* and *miR-25-3p* expression exhibited an inverse relationship, as did *miR-25-3p* and *miR-451a* in individual lesions. Over-expression of *miR-451a* in 12Z cells resulted in down-regulation of *miR-25-3p*, while up-regulation of *miR-25-3p* resulted in down-regulation of PTEN protein expression. Conclusions: By assessing individual endometriotic lesion expression, we discovered an inverse relationship between *miR-451a*, *miR-25-3p* and *PTEN*, while in vitro cell transfection studies suggest that *miR-451a* may regulate PTEN expression via modulating *miR-25-3p*.

## 1. Introduction

Endometriosis is a chronic, benign inflammatory gynecological disease, in which endometrial glands and stroma establish in the peritoneal cavity [1,2]. Although several theories on its etiology have been proposed, the theory of retrograde menstruation is the best well-established model [3]. According to this theory, with each menstrual cycle, in addition to normal antegrade menstruation, there is some degree of reverse menstruation by which shed endometrial tissue that contains endometrial stroma, epithelium and stem cells is deposited via the fallopian tubes into the pelvic cavity, establishing ectopic lesions. Although reverse menstruation is now believed to occur in the majority of women who exhibit menstrual cycles [4], endometriosis occurs in approximately 10% of women of reproductive age. Characterized by pelvic pain and infertility, endometriosis reduces quality of life and is associated with significant comorbidities [1,2]. The pathophysiology of endometriosis is complex as it is considered a multifactorial disease, which is thought to occur due to abnormal hormonal, epigenetic, genetic, and immunologic/inflammatory pathways, among others [5]. Further, because endometriosis is established when patients present for diagnosis, it is difficult to establish a cause and effect relationship between those factors that are misexpressed in the ectopic lesion tissue compared to eutopic endometrial tissue. In addition to early studies, which evaluated misexpressed mRNA transcripts between tissue, more recent studies have focused on the post-transcriptional regulation of these mRNAs by small non-coding RNAs known as microRNAs (miRNAs) [6,7,8]. However, these studies also are limited by the inability to establish a cause and effect relationship between those miRNAs that are misexpressed, which may lead to development/progression of the disease, and those which may be misexpressed as a result of the disease.

We previously reported in human [9,10] as well as in experimental endometriosis animal models [10,11], that *miRNA-451a* (*miR-451a*) is misexpressed in endometriotic lesion tissue where it may contribute to modulating cell survival and disease progression. Specifically, we proposed that the elevated *miR-451a* curtails lesion survival through post-transcriptional regulation of cell survival factors and this postulate is supported by our previously published body of work [9,10,11,12]. In our initial report [9], we found that overall expression of *miR-451a*, as well as that of the tumor suppressor, phosphatase and tensin homologue (*PTEN*), were both elevated in endometriotic lesion tissue compared to matched eutopic endometrial tissue. Our observation of increased *PTEN* transcript expression in endometriotic lesion tissue may be interpreted to suggest that this increase may be a response to limit lesion survival as PTEN is a known tumor suppressor [13,14]. However, our observation is in contrast to reports subsequently published. Specifically, *PTEN* mRNA was reported to be reduced in endometriotic lesion tissue [15,16,17], especially those which exhibit progesterone resistance [16], which would favor a proliferative phenotype. While these latter observations support the notion that reduced levels of PTEN in endometriosis may play a role in survival of the ectopic lesions, we were intrigued by our observation that PTEN was elevated in our study, which utilized a much larger sample size compared to the aforementioned studies [15,16,17]. Additionally, elevated PTEN expression and elevated *miR-451a* expression may suggest another mechanism by which this miRNA may limit endometriotic lesion survival. To begin to dissect the mechanisms that may contribute to elevated *PTEN* expression in endometriotic lesion tissue, we conducted the following series of experiments. 

## 2. Results

### 2.1. Endometriotic Lesion Expression of PTEN Transcript

**Figure 1:**
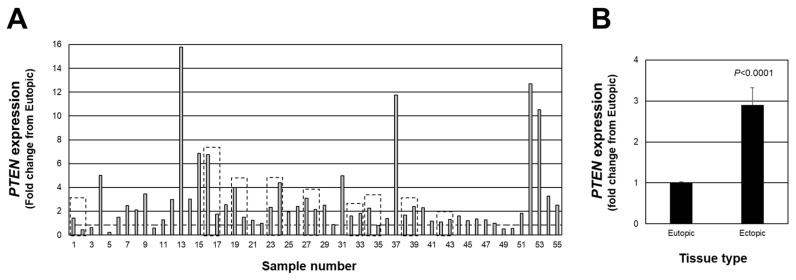
Fold change in *PTEN* expression in peritoneal endometriotic lesions. Data are expressed as the fold change from eutopic endometrial levels for each lesion (**A**) as well as the overall average fold change (**B**). Broken line in (**A**) depicts a fold-change of 1.0 which is equal to the expression in eutopic endometrial tissue. Boxed areas indicate lesions obtained from the same patient at the time of surgery.

We first examined individual lesion *PTEN* expression in red peritoneal lesions (a total of 55 lesions from 46 patients). We first assessed individual lesion expression expressed as a fold change from matched eutopic endometrium for each lesion (Figure 1A). Of the total 55 lesions, 45 lesions expressed a fold change above the expression level of matched eutopic endometrium (>1.0-fold with highest fold change of 15.76), while 10 of the 55 expressed a fold change below 1.0 (lowest value was 0.24). When the data were expressed as the average *PTEN* fold change from matched eutopic endometrium (Figure 1B), a significant (*p* < 0.0001) 2.9-fold increase was detected. These findings highlight the necessity to assess individual lesion expression of *PTEN* to thoroughly understand how this (and other cell survival factors) may change in endometriotic lesions as they progress from an active to less active lesion. This, in turn, would provide additional insight into the pathogenesis and pathophysiology of endometriosis.

### 2.2. Endometriotic Lesion Expression of miR-25-3p and Relationship with PTEN

**Figure 2:**
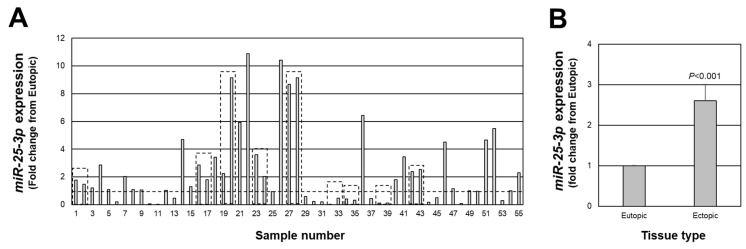
Fold change in *miR-25-3p* expression in peritoneal endometriotic lesions. Data are expressed as the fold change from eutopic endometrial levels for each lesion (**A**) as well as the overall average fold change (**B**). Broken line in (**A**) depicts a fold change of 1.0 which is equal to the expression in eutopic endometrial tissue. Boxed areas indicate lesions obtained from the same patient at the time of surgery.

As mentioned earlier, associated with a net increase in *PTEN* expression in endometriotic lesion tissue, we found an increase in *miR-451a* expression. Interestingly, *miR-451a* is not predicted to target the 3’ UTR of *PTEN* [18,19,20]. However, *PTEN* has been validated as a target of *miR-25-3p* [21,22,23] and has been reported to be misexpressed in endometriotic lesion tissue [24,25,26]. Therefore, we next assessed the expression of *miR-25-3p* in each of the same lesions in which we assessed *PTEN* expression. *miR-25-3p* expression ranged from approximately 11-fold higher (sample number 22) to 1700-fold lower (sample number 32) in endometriotic lesion tissue compared to matched eutopic endometrium (Figure 2A), with an overall increase in expression of 2.6-fold above levels in eutopic endometrium (Figure 2B; *p* < 0.001). *miR-25-3p* expression was greater than 1.0-fold higher than eutopic endometrial tissue in 34 of 55 lesions and equal to or less than 1.0-fold in 21 of 55 lesions. 

**Figure 3:**
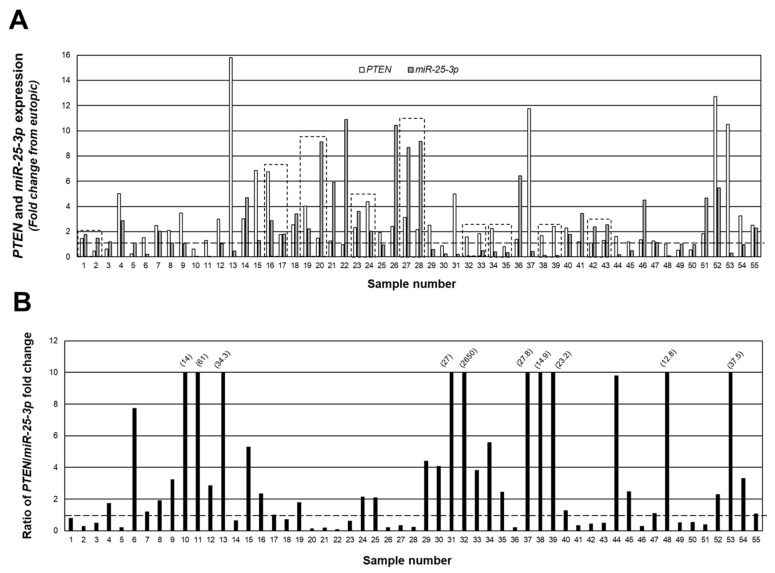
Individual peritoneal lesion expression and ratio of *PTEN*/*miR-25-3p* expression. The fold change from matched eutopic endometrium for *PTEN* and *miR-25-3p* were expressed for each lesion (**A**) as well as the ratio of *PTEN*/*miR-25-3p* expression (**B**) to gain insight into the ratio/relationship between the two in individual lesions. Numbers in parenthesis reflect actual ratio values for samples number 10, 11, 13, 31, 32, 37, 38, 39, 48, and 53. These ratios were capped at 10 to allow depiction of ratios below 1.0 (indicated by broken line). Boxed areas indicate lesions obtained from the same patient at the time of surgery.

Figure 3A depicts a merging of the data from Figure 1 and Figure 2 to show the relationship (fold change expression) between *PTEN* and *miR-25-3p*, while in Figure 3B, we present the data as the ratio of *PTEN*/*miR-25-3p* expression in individual lesions. Despite the fact that all lesions were classified as red (active) peritoneal lesions, there were clear differences in the level of expression and ratio of *PTEN*/*miR-25-3p*, ranging from a ratio of 2650 *PTEN*/*miR-25-3p* (sample #32; Figure 3B) to 0.09 *PTEN*/*miR-25-3p* (sample #22; Figure 3B). Of the 55 lesions, in 34 of these lesions, the level of *PTEN* expression exceeded that of *miR-25-3p*, while in the remaining 21 lesions, *miR-25-3p* expression exceeded that of *PTEN*. These data clearly indicate that despite a similar appearance and classification of lesion type, there were clear differences in expression of *PTEN* and *miR-25-3p*. 

### 2.3. Relationship between miR-451a and miR-25-3p In Vivo

**Figure 4:**
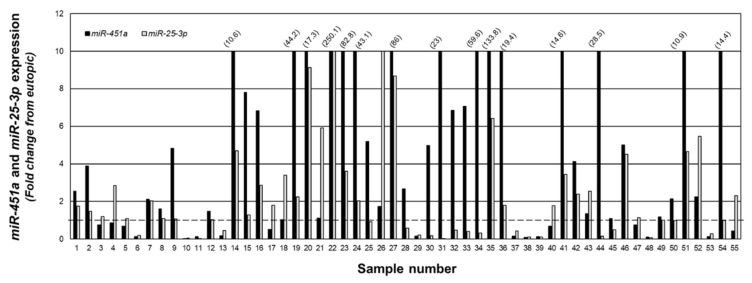
Fold change in *miR-451a* and *miR-25-3p* expression in peritoneal endometriotic lesions. Data are expressed as the fold change from eutopic endometrial levels for *miR-4541a* and *miR-25-3p* in the same sample. Numbers in parenthesis reflect actual fold change increase for *miR-451a* in specified sample number. The fold change was capped at 10 to allow depiction of ratios below 1.0 (indicated by broken line).

To examine the relationship between *miR-25-3p* and *miR-451a*, we quantitated expression of both miRNAs in the same specimens and present the data as fold change from matched eutopic endometrium (Figure 4). In 36 of the 55 lesion samples, *miR-451a* expression was greater than that of *miR-25-3p*, while *miR-25-3p* expression was greater than that of *miR-451a* in the remaining 19 samples.

### 2.4. miR-451a Regulatoin of miR-25-3p In Vitro

**Figure 5:**
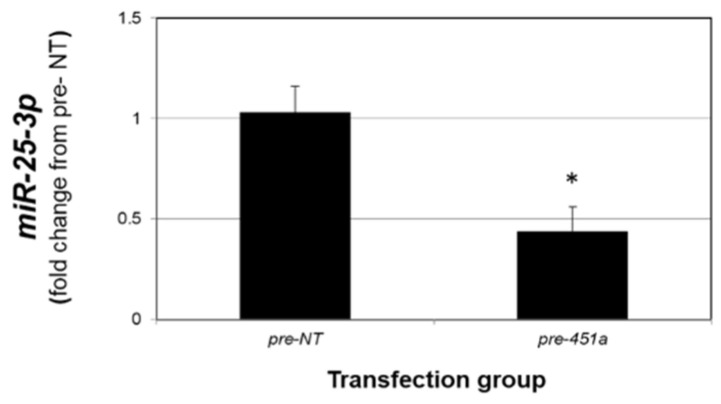
*miR-451a* decreases *miR-25-3p* expression in endometriotic epithelial 12Z cells. Asterisk indicates statistically significant (*p* < 0.01) reduction compared to pre-NT transfected cells. N = 3 replicates.

As the data presented in Figure 4 support an inverse relationship between *miR-451a* and *miR-25-3p*, we next explored if elevated levels of *miR-451a* could suppress *miR-25-3p.* Compared to 12Z cells transfected with non-targeting mimics (pre-NT), transfection of 12Z cells with *miR-451a* mimics (*pre-miR-451a*) resulted in a significant reduction in *miR-25-3p* levels (Figure 5). These data could be interpreted to suggest that elevated levels of *miR-451a* may also limit lesion survival by modulating PTEN expression via decreasing *miR-25-3p* expression, as *PTEN* is a validated target of *miR-25-3p*.

### 2.5. miR-25-3p Down-Regulates PTEN Protein In Vitro

**Figure 6:**
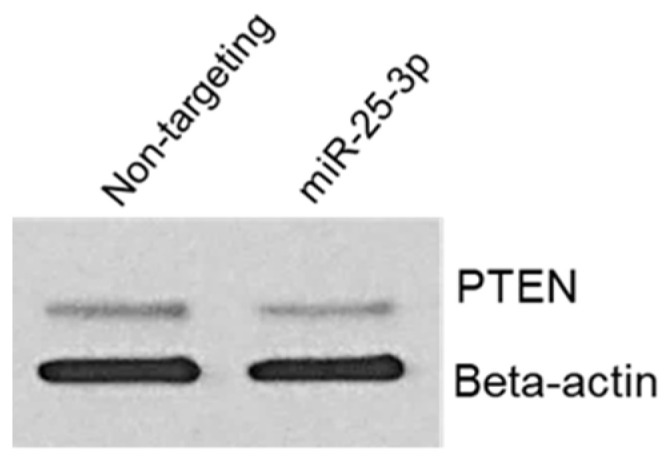
miR-25-3p decreases endometriotic epithelial 12Z cell PTEN protein expression.

To confirm that *miR-25-3p* regulates PTEN protein expression, we transfected 12Z cells with either non-targeting control mimics or *miR-25-3p* mimics. Cells were then harvested for protein and Western blotting and PTEN was conducted. As depicted in Figure 6, *miR-25-3p* transection resulted in a decrease in PTEN protein expression but had no effect on expression of beta-actin, which was used as a loading control. This experiment was repeated with two different cell passages, both yielding similar results. This observation is in accord with similar studies, in which *miR-25-3p* was confirmed to decrease PTEN protein expression.

## 3. Discussion

miRNAs have been proposed to play a role in the pathophysiology of endometriosis [6,7,8]. There is limited information on miRNA profiles comparing peritoneal endometriotic lesions to that of matched eutopic endometrium. The first report by Ohlsson-Teague and associates [27] utilized four specimens from the follicular and three from the secretory stages of the menstrual cycle, comparing ectopic peritoneal lesion expression to that of eutopic endometrium from the same patient. miRNA expression was assessed by microarray hybridization and three up- and three down-regulated miRNAs were confirmed by qRT-PCR. More recently, two additional studies assessed ectopic lesion miRNA expression but the ectopic lesion type was not specified [15,25]. In contrast, our study specifically assessed red peritoneal lesions, which are considered early, active lesions. Secondly our sample size of 55 lesions is, to the best of our knowledge, one of the largest sample sizes reported, which increases scientific rigor. Thus, we feel that while initial studies identified potentially important miRNAs in endometriosis pathophysiology, limited sample sizes may prohibit a full appreciation for miRNA profiles in endometriotic lesions.

Our study also reveals that while all lesions appeared similar and were classified as red peritoneal lesions, we observed stark contrasts in the expression level of each endpoint assessed. The majority of endometriosis research reports changes in expression of study endpoints (mRNA, protein, miRNA, etc.) as an overall fold change from the control tissue. As can be seen in our study (Figure 1 and Figure 2), this may be misleading. For example, higher expression of PTEN, a known tumor suppressor [13,14], in active endometriotic lesion tissue would contradict such a role, as one would expect its expression to be lower compared to control eutopic endometrium (as has been reported by others [15,16,17]). However, when individual lesion expression is examined, a full spectrum of lower, higher, or similar expression levels to that of eutopic endometrium levels of *PTEN* expression is evident. We interpret these results to suggest that the most active lesions express the lowest levels of PTEN and as the lesion progresses towards a less active form, PTEN levels gradually increase, leading to the suppression of lesion proliferation.

Along these lines, our observation that overall average expression of both *PTEN* and *miR-25-3p* are elevated in endometriotic lesion tissue also seems to be contradictory if *miR-25-3p* down-regulates *PTEN* expression, as has been reported in the literature. However, these observations emphasize the need to evaluate endometriotic lesions on an individual basis. Data presented in Figure 3 demonstrate an inverse relationship between miRNA and mRNA target, such that high levels of expression of *miR-25-3p* are associated with lower levels of *PTEN* transcript, and we postulate that these would be very early established lesions, while those red lesions, which express lower *miR-25-3p* and higher *PTEN* expression, would be “older” lesions, which are more likely to progress to an intermediate blue/black lesion. These observations support our cell transfection study (Figure 6), in which increased *miR-25-3p* expression is associated with decreased PTEN protein expression, and are in agreement with similar results obtained in retinoblastoma [21], esophageal cancer cells [22], and multiple myeloma cells [23].

Our observation that *miR-451a* and *miR-25-3p* expression in individual lesions show an inverse relationship, coupled with the fact that *miR-451a* does not [18,19,20], but *miR-25-3p* does regulate *PTEN* expression [18,19,20,21,22,23], leads us to further explore the relationship between these two miRNAs. miRNA:miRNA interaction is a form of self-regulation of miRNA expression, which can occur through either direct, indirect, or global interactions [28,29]. Direct miRNA:miRNA interactions occur when a mature miRNA binds with another miRNA, either targeting the mature or pri-miRNA. Examples of direct targeting include *miR-424* and *miR-503* directly binding with the pri-form of *miR-9* [30] and *miR-709* binding with pri-*miR15a*/*16-1* [31], while *miR-107* has been reported to bind with the mature form of let-7 [32]. In our study, we found that forced expression of *miR-451a* led to a reduction in *miR-25-3p*, which, to the best of our knowledge, is the first report of a potential regulation by *miR-451a*. While it is uncertain if this regulation occurs via miRNA:miRNA interaction, and if so, by what mechanisms, further detailed studies are warranted to examine this mechanism in endometriosis. 

In addition, further studies should also explore the potential of *miR-451a* regulation of other miRNAs, which may be relevant in endometriosis pathophysiology. *miR-451a* is elevated in sera from women with endometriosis and has been proposed as a potential non-invasive biomarker for disease detection [12,33,34]. Based upon the results of our experimental endometriosis animal model studies [10,11], we propose that these elevated levels of *miR-451a* curtail lesion survival. This conclusion is based upon our findings that in baboons with experimentally induced endometriosis, serum *miR-451a* levels rise in a cyclic fashion after induction of the disease [11], which suggested to us that elevated serum levels were a result of the presence of ectopic lesions/endometriosis. Additional studies by us [10] revealed that induction of endometriosis in a mouse model using *miR-451a*-deficient tissue to establish ectopic lesions provided insight into progression of lesion *miR-451a* content. Specifically, lesion tissue levels of *miR-451a* increased in a time-dependent fashion post induction of the disease and these elevated levels inversely correlated with lesion tissue expression of the pro-survival factor macrophage migration inhibitory factor, or Mif [10]. Thus, elevation of *miR-451a* may limit lesion survival by not only directly down-regulating expression of pro-survival factors, such as Mif, but also via miRNA:miRNA interaction, leading to the modulation of tumor suppressors, such as PTEN. 

**Figure 7:**
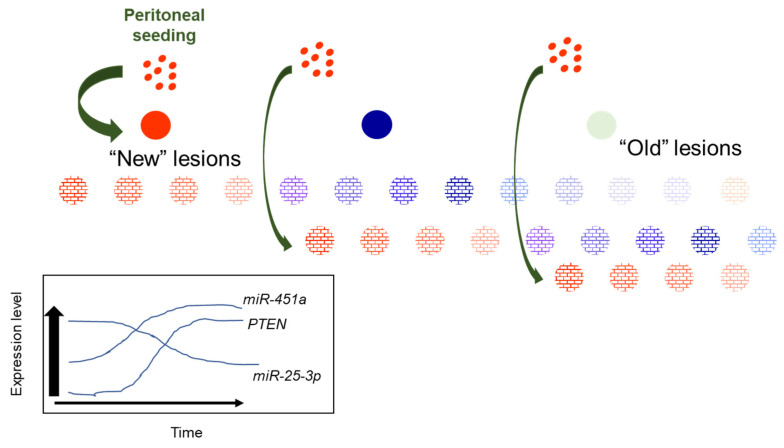
Proposed working model for heterogeneity of *miR-451a, miR-25-3p* and *PTEN* expression in red peritoneal lesions as they transition to regression.

Based upon results from the current study, as well as our prior studies on *miR-451a*, we postulate that increasing levels of *miR-451a* may curtail lesion survival and growth at multiple levels. As previously reported [9], *miR-451a* may regulate MIF, which has been proposed to contribute to lesion survival. From the results of the current study, we propose that *miR-451a* may also modulate the expression of other miRNAs, such as *miR-25-3p*, which, in turn, may restore PTEN expression to limit lesion survival. The potential regulation of *miR-25-3p* by *miR-451a* is intriguing and warrants more detailed mechanistic studies. Based upon the data presented in this report, and in conjunction with our prior studies [9,10,11], we propose the following working model (Figure 7). 

New, red endometriotic lesions develop with each episode of reverse menstruation and seeding of the peritoneal cavity. In these new lesions, expression of *miR-451a* and *PTEN* is low while that of *miR-25-3p* is high. With each subsequent episode of peritoneal seeding, “new” lesions are established, characterized by low *miR-451a* and *PTEN* and elevated *miR-25-3p* expression. As lesions “age”, their *miR-451a* and *PTEN* levels increase while *miR-25-3p* levels decrease to suppress lesion survival, eventually leading to lesion regression. This eventually leads to transition from “new” active red lesions to intermediate (blue/black) then fibrotic/regressing inactive lesions. The proposed model may also explain why some lesions regress completely without progressing to a fibrotic lesion. Further, if one focuses on a hypothetical case in which a patient may present with 12 red lesions (Figure 7), which have been established over the course of subsequent episodes of retrograde menstruation, one can see from our schematic that it would be possible to detect different levels of *miR-451a*, *miR-25-3p*, and *PTEN*, based upon the “age” of the lesion (the duration for which that specific lesions has been in the pelvic cavity). Thus, random sampling of red peritoneal, active lesions, although they all appear similar, leads to heterogeneity of results (*miR-451a*, *miR-25-3p*, *PTEN*, etc. expression).

## 4. Materials and Methods

### 4.1. Human Subjects and Tissue Acquisition

The study was approved by the institutional review boards of both the University of Kansas Medical Center and Cleveland Clinic. Written informed consent was obtained prior to surgical removal of endometriotic lesion tissue and endometrial biopsies. Women with endometriosis who presented with pelvic pain due to failed previous endometriosis treatment and were undergoing surgical removal of endometriotic lesion tissue were enrolled. No subjects had taken GnRH analogs or hormonal therapy within 3 months prior to surgery. A total of 46 subjects (ages 21 to 45) were enrolled from which 55 red, peritoneal endometriotic lesions were obtained. In 9 of these patients, we were able to obtain 2 separate lesions from different sites on the peritoneum. Endometriosis was classified as stage I/II or stage III/IV according to the revised (1996) American Society for Reproductive Medicine guidelines [35]. Of the stage I/II subjects (N = 18), seven were in the proliferative stage of the menstrual cycle and eleven were in the secretory stage while the remaining endometriosis in the remaining 28 subjects was classified as stage III/IV with eleven in the proliferative and seventeen in the secretory stage of the menstrual cycle. For this study, we utilized banked mRNA from specimens obtained in our previous study [10] as well as additional patients assessing only red peritoneal lesions and matched eutopic endometrial tissue. 

### 4.2. mRNA Isolation and qRT-PCR

Quantitative real-time PCR (qRT–PCR) was performed as previously described by us [9,10,11,36]. Briefly, total RNA was isolated using Tri-Reagent (Sigma Chemical Co., St. Louis, MO, USA) according to recommendations of the manufacturer. Total RNA (1 µg in 20 µL) was reverse transcribed using reverse transcription (RT) kits (Applied Biosystems; Foster City, CA, USA) following the manufacturer’s protocol. Primers for phosphatase and tensin homolog (PTEN) were designed using Primer-Blast and synthesized by Integrated DNA Technology (IDT, Coralville, IA): human PTEN (NM_000314): forward, 5′-AAGACATTATGACACCGCCAAA-3′ and reverse, 5′-GTGGGTTATGGTCTTCAAAAGGA-3′. Resulting material was then used for independent qRT–PCR. qRT–PCR was carried out on an Applied Biosystems HT7900 Sequence Detector. To account for differences in starting material, human 18S primers (catalog #4310893E; ThermoFisher Scientific, Waltham, MA, USA) were used.

*miR-25-3p* expression was quantitated using a specific miRNA assay kit from Applied Biosystems (Waltham, MA, USA). Total RNA (250 ng in 5 µL) was reverse transcribed using RT kits (Applied Biosystems) following the manufacturer’s protocol with the following modifications. Briefly, miRNAs were reverse transcribed in a single reaction using 2 µL of each miRNA specific 5X RT primers. Resulting material was then used for independent qRT–PCR for each miRNA. To normalize for starting material, a reverse snRNA U58 was included in the miRNA RT reactions and qRT–PCR of U58 was performed. qRT–PCR reactions were completed on a 7900 HT Sequence Detection System (Applied Biosystems). All samples were run in triplicate and the average value used in subsequent calculations. The 2-delta-delta CT method was used to calculate the fold change values among samples as previously described by our group [9,10,11,36].

### 4.3. Cell Culture of Endometriotic Epithelial 12Z Cells and Transfection

The endometriotic epithelial cell line, 12Z, was obtained from Dr. Linda Griffith (Massachusetts Institute of Technology, Cambridge, MA, USA). Cell culture was conducted following the general approach as previously described [9,36]. Briefly, cells were cultured in phenol red-free Dulbecco’s Minimum essential medium (DMEM)/Ham’s F12 (Fisher Scientific, Pittsburgh, PA, USA) + 10% charcoal-stripped FBS (Atlanta Biologicals, Atlanta, GA, USA)  +  Pen-Strep (Life Technologies, Carlsbad, CA, USA) in T75 flasks and seeded at 1 × 10^6^ cells/mL of media until approximately 90% confluency. Cells were then passed and plated in 6-well plates at a density of 1 × 10^5^ cells/mL in DMEM/Ham’s F12 media lacking FBS and Pen-Strep. The next day, cells were transfected as described below.

To assess the impact of *miR-451a* and *miR-25-3p*, 12Z cells were separately transfected with mimics (ThermoFisher) for each miRNA or a non-targeting (NT) mimic which was used as a negative control (30 nM final concentration of each mimic). Briefly, 12Z cells were cultured in phenol red-free DMEM:F12 supplemented with 10% charcoal-stripped FBS, penicillin, and streptomycin. Cells were transfected at 50% confluency using Lipofecateamine-2000 transfection agent according to recommendations of the manufacturer. Twenty-four to forty-eight hours after transfection, *miR-451a, miR-25-3p* and *PTEN* transcript expression were assessed by qRT-PCR while PTEN and beta-actin (normalizing control) protein levels were assessed by Western blotting as described below.

### 4.4. Western Analysis 

Total protein was extracted from 12Z cells using RIPA buffer (1X RIPA, Catalog #9806, Cell Signaling Technologies (CST), Danvers, MA, USA). Protein concentration in each sample was determined using the Bio-Rad Protein Assay ((Catalog 3500-0006), Bio-Rad Laboratories, Richmond, CA, USA). The same amount of protein (30 μg) was subjected to 12% Bis(2-hydroxyethyl)amino-tris(hydroxymethyl)methane (*w/v*) gel electrophoresis and electroblotted onto PVDF membranes (Invitrogen). PTEN (9188; 1:1000; CST) and donkey, anti-rabbit secondary antibody (catalog #NA934V; 1:20000; GE Healthcare/Fisher Scientific, Pittsburgh, PA, USA) were used. Stripping and re-probing for β-actin (ab8227; 1:10,000; Abcam, Cambridge, MA, USA) were conducted to normalize PTEN protein expression levels. 

### 4.5. Statistical Analysis

miRNA and mRNA levels were first separately assessed within stage of endometriosis (stage I/II vs. stage III/IV in endometriosis subjects) and among stage of menstrual cycle. As no significant differences among their expression could be attributed to stage of endometriosis or stage of menstrual cycle, data were pooled and analyzed as eutopic endometrial tissue compared to endometriotic lesion tissue. Comparisons between two groups were made using two-tailed unpaired student t-tests with two-sample unequal variance when warranted.
